# Could Vaccination against COVID-19 Trigger Immune-Mediated Inflammatory Diseases?

**DOI:** 10.3390/jcm13164617

**Published:** 2024-08-07

**Authors:** Aikaterini I. Liakou, Andreas G. Tsantes, Eleni Routsi, Efthymia Agiasofitou, Magdalini Kalamata, Evangelia-Konstantina Bompou, Konstantina A. Tsante, Soultana Vladeni, Eleni Chatzidimitriou, Ourania Kotsafti, George Samonis, Stefanos Bonovas, Alexander I. Stratigos

**Affiliations:** 1 1st Department of Dermatology-Venereology, “Andreas Sygros” Hospital, National and Kapodistrian University of Athens Medical School, 16121 Athens, Greece; routsie@gmail.com (E.R.); efi.agiasofitou@yahoo.com (E.A.); magda.kalamata@gmail.com (M.K.); valentina.bompou@gmail.com (E.-K.B.); vladeni@otenet.gr (S.V.); elinahatzidim@gmail.com (E.C.); raniakotsafti@gmail.com (O.K.); alstrat2@gmail.com (A.I.S.); 2 Laboratory of Haematology and Blood Bank Unit, “Attikon” Hospital, National and Kapodistrian University of Athens Medical School, 12462 Athens, Greece; andreas.tsantes@yahoo.com (A.G.T.); ktsante@yahoo.com (K.A.T.); 3 Microbiology Department, “Saint Savvas” Oncology Hospital, 11522 Athens, Greece; 4 Department of Medicine, University of Crete, 71500 Heraklion, Greece; samonis@med.uoc.gr; 5 Department of Oncology, Metropolitan Hospital, 18547 Athens, Greece; 6 Department of Biomedical Sciences, Humanitas University, 20072 Pieve Emanuele, Milan, Italy; sbonovas@gmail.com; 7 IRCCS Humanitas Research Hospital, 20089 Rozzano, Milan, Italy

**Keywords:** immune-mediated inflammatory diseases, COVID-19, vaccine, flare, onset, hidradenitis

## Abstract

Exacerbations and new onset of immune-mediated inflammatory diseases, such as psoriasis and hidradenitis suppurativa, have been reported following COVID-19 vaccination. In patients with hidradenitis suppurativa, recent studies have shown that those who received mRNA vaccines were 3.5 times as likely to develop flares following vaccination compared to patients who received non-mRNA vaccines, indicating that mRNA COVID-19 vaccines are associated with hidradenitis suppurativa flares. Similar findings have been found in other studies evaluating the association between COVID-19 vaccines and other immune-mediated inflammatory diseases such as psoriasis, atopic dermatitis, lichen planus, and alopecia areata. However, further research is warranted in larger populations to validate these findings.

## 1. Introduction

Severe acute respiratory syndrome coronavirus 2 (SARS-CoV-2) spread rapidly throughout the world in 2019–2020, resulting in the coronavirus disease (COVID-19) pandemic [1]. The development of effective vaccines against the SARS-CoV-2 virus was a significant breakthrough in the battle against the pandemic. Different types of vaccines were developed using various vectors and methods of production, such as messenger RNA (mRNA) vaccines (BioNTech/Pfizer, Moderna), viral vector vaccines (AstraZeneca/Oxford, Janssen/Johnson & Johnson, CanSino), and inactivated or weakened virus vaccines (Sinovac-Coronavac, Sinopharm, Bharat, Valneva) [2].

Immune-mediated inflammatory diseases (IMIDs) are a large group of diseases, whose etiology involves an altered immune response to environmental triggers in genetically susceptible individuals. There are several concerns regarding COVID-19 vaccination in patients with IMIDs. Concerns about potential exacerbation of disease symptoms, adverse events, and the impact on disease activity and treatment response have been reported in the international literature [3,4,5,6,7]. Existing evidence suggests that COVID-19 vaccination may affect the course of IMIDs and lead to temporary disease exacerbation [8,9]. Patients with IMIDs may also have a decreased immune response following vaccination due to the disease itself or due to the treatment, especially those who are on biologic therapy [9].

Th1-type cytokines are known for generating proinflammatory responses that are crucial for eliminating intracellular parasites and sustaining autoimmune responses, with interferon gamma being the primary Th1 cytokine. On the other hand, Th2-type cytokines, such as interleukins 4, 5, and 13, promote IgE production and eosinophilic responses in atopic conditions. Interleukin-10, another Th2 cytokine, primarily exerts anti-inflammatory effects. An overactive Th2 response can inhibit the Th1-mediated microbicidal activity. Thus, an optimal immune response involves a well-balanced Th1 and Th2 activity, tailored to the specific immune challenge [10].

In the context of IMIDs, specific cytokines play pivotal roles. Specifically, psoriasis is predominantly Th1/Th17-mediated. Key cytokines include IFN-γ, IL-17, IL-22, and TNF-α. MRNA vaccines can potentially enhance Th1 responses, leading to increased production of these cytokines and exacerbation of psoriasis symptoms. HS is associated with dysregulation of both Th1 and Th17 pathways, with key cytokines including TNF-α, IL-1β, and IL-17. The heightened innate immune activation by mRNA vaccines might contribute to increased levels of these proinflammatory cytokines, potentially triggering flares [11,12].

The underlying mechanisms by which vaccination may trigger or worsen IMIDs remain unclear while it is crucial to differentiate between vaccine-related reactions and natural disease progression [13,14]. A few authors have reported that COVID-19 vaccines result in inhibition of the T-helper 2 cell pathway and the induction of the T-helper 1 cell pathway. This could explain the role of COVID-19 vaccines in IMID flares, as these mechanisms have been widely established as the basis for the development of disease symptoms. Moreover, it has been hypothesized that overstimulation of the innate immune system caused by COVID-19 vaccines may lead to increased disease activity and worsen clinical manifestations in susceptible patients [15,16]. Given the complexities of the immune system and the variability in individual responses, it is essential to continue researching the specific mechanisms by which COVID-19 vaccines interact with the immune system, particularly in the context of IMIDs. The exact mechanisms by which COVID-19 vaccines influence the Th1 and Th2 pathways require further investigation. This review aims to consolidate and present all the recent evidence regarding the association between COVID-19 and IMIDs, focusing on the impact of COVID-19 disease and its vaccines on the natural history of IMIDs and vice versa the impact of IMIDs and their associated treatments on the efficacy of COVID-19 vaccines.

## 2. COVID-19 Vaccines and Hidradenitis Suppurativa

A retrospective observational study was recently performed including 250 patients with hidradenitis suppurativa (HS) who were vaccinated for COVID-19 disease. HS is a chronic inflammatory skin condition, primarily affecting the apocrine gland-bearing areas of the body, such as the axillae, inguinal, and anogenital regions [17,18,19]. Given the immunological dysregulation seen in HS, questions have arisen regarding the safety and efficacy of COVID-19 vaccination in individuals with HS [20,21,22,23]. The results of this study indicated that mRNA vaccines are associated with a higher risk of HS flares compared to non-mRNA vaccines. Specifically, it was shown that patients who had received mRNA vaccines were 3.5 times as likely to develop HS flares following vaccination compared to patients who had received non-mRNA vaccines. Although this finding is notable, this alone does not justify restricting the use of mRNA vaccines. The benefits of vaccination in preventing severe COVID-19 far outweigh the risks associated with potential flares, which can be managed with appropriate medical care. Another interesting finding was that patients who were on biological treatment for HS were less likely to develop HS flares following vaccination, indicating that biological treatment may have a protective role against HS flares. The assertion that patients on systemic medications within a year of vaccination are not at higher risk and that vaccination is not contraindicated is particularly relevant. This perspective supports the continued use of mRNA vaccines among HS patients on systemic treatments, ensuring they are not unjustly excluded from the protective benefits of vaccination. Further research is warranted in larger populations to validate these findings.

Only a few small studies investigated the impact of COVID-19 vaccination on patients with HS. In a series of five patients, Martora et al. reported HS exacerbations following COVID-19 vaccination. These flares were controlled through antibiotic therapy or intralesional steroids. The authors of this study recommended that a second or third dose of the COVID-19 vaccine should not be contraindicated in patients who developed an HS flare after the first dose. This recommendation is also in line with the protocol of our department since the flares reported in our study were not considered a contraindication to further vaccination. Interestingly, all five patients regularly completed the vaccine course. The authors conclude that the mechanism is unknown, but it can be hypothesized that the COVID-19 vaccine may inhibit the T helper 2 cell pathway and at the same time promote the T helper 1 cell pathway [24]. In another study, Alexander et al. reported a case of new-onset HS in a patient after COVID-19 vaccination. This patient developed multiple abscesses in his axillary and groin areas 3 weeks following the second dose of the AstraZeneca COVID-19 vaccine. Interestingly, the manifestation of HS was incrementally more severe after the second vaccine dose than after the first dose, implicating immunologic memory in the pathogenesis of this presentation. The authors report that the AstraZeneca COVID-19 vaccine stimulates innate immune responses by involving multiple pattern recognition receptors, particularly Toll-like receptor 9; this could explain the correlation along with the timing of lesion appearance. Furthermore, metabolic syndrome was the key patient risk factor typically associated with spontaneous HS [25]. A retrospective study from Italy on patients with HS undergoing COVID-19 vaccinations enrolled 200 patients (92 men, 108 women; mean ± SD age of 39.6 ± 6.5 years). The mean duration of HS was 8.2 ± 4.3 years. Regarding ongoing HS treatment, 168 patients (84%) were treated with adalimumab, while the rest were treated with topical and/or systemic antibiotics (rifampicin and clindamycin). The authors did not compare the HS patients with those without HS. However, based on the data presented from the study, the authors concluded that the risk of COVID-19 vaccination-related AEs was not higher in patients with HS, even in those treated with biological drugs (adalimumab) [4].

According to the 10th International Conference, European Hidradenitis Suppurativa Foundation experts have prepared a consensus statement regarding anti-COVID-19 measures for HS patients. The experts propose vaccination for COVID-19 disease for HS patients without specific contraindications. Patients with HS should be encouraged to receive COVID-19 vaccination, considering the potential benefits of reducing severe illness from SARS-CoV-2. Specifically, individuals with metabolic syndrome, which is a group that is observed more often in HS and recognized as a high-risk group for COVID-19 complications, should also be encouraged to get vaccinated without delay. HS patients undergoing treatment with adalimumab can safely receive non-live virus COVID-19 vaccines. Treatment with adalimumab should not be interrupted, especially in moderate-to-severe HS. However, the initiation of HS treatment with treatments other than adalimumab and antibiotics should be carefully evaluated. Decisions regarding the continuation or adjustment of biological treatments in HS patients’ post-vaccination should be made in consultation with dermatologists or managing physicians, considering individual patient factors and disease management goals [21]. Another large-scale multicenter (TriNetX, Cambridge, MA, USA) retrospective cohort study by Pakhchanian et al. included vaccinated patients with HS and compared them to a control vaccinated cohort without HS. The study involved a sample of more than 3000 patients. The aim was to investigate the safety and efficacy of COVID-19 vaccines among patients with HS. The results showed that patients with HS are not at a significantly higher risk for any vaccine-related adverse outcomes. Furthermore, patients with HS on systemic medications within one year of being vaccinated had similar outcomes to those not receiving medications, demonstrating that the anti-inflammatory/immunosuppressive therapy should not be viewed as a contraindication to vaccination, as mentioned above [26].

Taking the aforementioned findings into consideration, clinicians should be aware of the potential for HS exacerbation following COVID-19 vaccination and monitor patients accordingly. Patients with a history of HS should be informed about the possibility of flares and advised on managing symptoms if they occur. Despite the rare risk of IMID exacerbation, the benefits of COVID-19 vaccination in preventing severe disease and controlling the pandemic far outweigh these risks. Individualized patient management and informed discussions are crucial. For HS patients, vaccination strategies should include pre-vaccination assessment to evaluate disease activity and optimize HS management before vaccination, and post-vaccination monitoring for signs of HS exacerbation. Symptomatic management is also crucial in the temporary adjustment of HS treatments to manage flare-ups. Of course, the above are not recommendations for clinicians but the authors’ conclusions drawn from the collected evidence. There is also a need for larger epidemiological studies to establish a clear causal relationship between COVID-19 vaccination and HS. Mechanistic studies should explore the specific immune pathways involved in vaccine-induced HS exacerbation.

## 3. COVID-19 Vaccines and Psoriasis

The association between COVID-19 vaccines and other autoimmune diseases has also been evaluated in several studies [27,28,29]. Patients with psoriasis are considered a priority for COVID-19 vaccination due to the frequently accompanying comorbidities and their treatment-induced immunosuppressive status. COVID-19 vaccination could be a triggering factor for psoriasis. Triggering of psoriasis by vaccines has been previously proposed with other vaccines, including against influenza, rubella, BCG, tetanus–diphtheria, and the pneumococcal polysaccharide vaccine. The process of skin lesion formation in psoriasis is complex. One of these mechanisms is the Koebner phenomenon, in which psoriatic lesions develop at the site of skin injury; this could possibly be associated with vaccination.

A variety of factors, such as the mycobacterial heat shock proteins in BCG and diphtheria toxoid in BCG, tetanus–diphtheria, and influenza vaccines, can induce IL-6 associated with the generation of Th17 cells. Adenovirus vectors, mRNAs, and virus-related proteins in some of the most frequently administered COVID-19 vaccines are known inducers of Th1 and Th17 responses. This process could also be mediated by the increased production of TNF-α and type I interferon by CD4+ T cells and plasmacytoid dendritic cells, which may participate in the activation of psoriasis [30].

Various psoriasis subtypes have been defined, including plaque, guttate, pustular, and nail psoriasis and psoriatic arthritis (PsA). Treatment modulatory agents, like interleukin IL-17 inhibitors, have the potential to compromise mucosal immunity, consequently elevating the susceptibility to upper and lower respiratory tract infections [8]. Furthermore, anti-tumor necrosis factor (anti-TNF) agents and other immunosuppressive medications, like methotrexate and cyclosporine, may also increase the susceptibility to pulmonary infections, including SARS-CoV-2. Another reason why vaccination is important is that psoriasis patients on immunosuppressive medications are at an increased risk for post-infectious complications, potentially emerging with more severe forms of SARS-CoV-2 infection [31]. A review summarizing evidence up to April 2023 reported a total of 98 cases, 81 of which involved flare-ups while the remaining 17 cases involved new onsets of psoriasis. The most common subtypes of psoriasis were plaque and guttate psoriasis. Erythrodermic, nail, and pustular psoriasis were reported more rarely. Regarding the type of anti-SARS-CoV-2 vaccine, most cases were attributed to the BioNTech/Pfizer COVID-19 vaccine. However, this last finding should be interpreted with caution considering that this specific type of vaccination was the most administered [30]. Burlando et al. aimed to evaluate if the patients treated with biologics have a lower risk of psoriasis flares after COVID-19 vaccination than other psoriatic patients. The findings indicated that patients undergoing biologic therapy had a reduced risk of psoriasis flares following COVID-19 vaccination compared to those not on biologics. Specifically, the study demonstrated that 33.3% of patients under biologic treatment experienced psoriasis flares post-vaccination compared with 66.6% of patients not receiving biologic therapy (*p* = 0.021) [32]. A systematic review of 43 studies estimated the incidence of new-onset or flares of psoriasis following COVID-19 vaccination. It was shown that most psoriatic episodes occurred after vaccination with mRNA vaccines, while the second dose was the one most frequently associated with these episodes. The onset of these episodes ranged from 2 to 21 days in the new-onset group, and from 1 to 90 days in the flare group [30]. These findings are in line with our results since we also found that mRNA vaccines were associated with a higher incidence of HS flares, while most HS flares occurred after the second vaccination dose. Another systematic review by Potestio et al. including 49 studies and 134 patients reported that almost 20% were related to new-onset psoriasis. This systematic review stated that the duration of psoriasis may not be a predisposing factor for a psoriasis flare-up following COVID-19 vaccination. In contrast to our study, Potestio reported that the exacerbation of psoriasis or new-onset psoriasis occurrence has been linked to all COVID-19 vaccines, including mRNA vaccines, such as those developed by Moderna and BioNTech/Pfizer. Psoriasis onset was reported following the first, second, and third vaccine doses, with the second dose predominantly linked to psoriasis flares. The authors also reported in the case of non-COVID-19 vaccines, new-onset psoriasis occurrences are rare, while the guttate form was the most frequent psoriasis subtype that occurred after vaccination, different from COVID-19 vaccines [33]. In another study including 89 psoriatic patients who received inactivated (CoronaVac) or mRNA (BioNTech/Pfizer) COVID-19 vaccines, psoriatic flares occurred in 7% (6/89) of the population. Like the previous study, most flares were observed following the mRNA vaccine (5/6, 83%). Moreover, mRNA vaccines were associated with a higher incidence of adverse events following vaccination, although these adverse events were not severe. Finally, the authors of this study reported that psoriatic patients who were on biologic agents or methotrexate developed similar responses to the mRNA vaccine compared to those who were not receiving such treatment, while the response to the inactivated vaccine was weaker in these patients [34].

## 4. COVID-19 Vaccines and Other Autoimmune Diseases

### 4.1. Lichen Planus

Lichen planus (LP) is an inflammatory disorder of the skin and mucous membranes with no known cause. LP is an idiopathic disease. Although its pathogenesis is not fully understood, it appears to represent a T cell-mediated autoimmune disease. The prevailing theory is that exposure to an exogenous agent such as a virus, drug, or contact allergen causes alteration of epidermal self-antigens and activation of cytotoxic CD8+ T cells [35]. According to Martora et al., thirteen cases of new-onset disease have been documented in the literature up to April 2023, while worsening of LP following COVID-19 vaccination has been reported in three cases. The underlying mechanism remains unclear; similar reports of LP onset have been noted after other vaccinations, such as hepatitis B virus (HBV). Researchers speculate that vaccination may trigger a Th1 cell response and subsequent release of various cytokines, potentially influencing the onset of this condition [31]. One hypothesis is that molecular mimicry is responsible for triggering the autoimmune CTL and Th1 responses that mediate LP in both infection and vaccination. The SARS-CoV-2 antigen has demonstrated cross-reactivity with multiple endogenous human antigens, including those found on the basal keratinocytes of the epidermis [31]. Some attribute this antigen cross-reactivity to genetic similarities or shared epitopes.

### 4.2. Atopic Dermatitis

Atopic dermatitis (AD) is a chronic inflammatory disease that causes itchy skin with a subsequent psychosocial impact on patients and family members. AD is the most common chronic inflammatory skin disease. There are very few reports in the literature linking atopic dermatitis and COVID-19 vaccination. AD is primarily associated with a skewed Th2 immune response, characterized by the overproduction of Th2 cytokines such as IL-4, IL-5, and IL-13. These cytokines promote IgE production and eosinophil activation and contribute to the inflammatory milieu [36]. According to an observational multicenter study from Korea, the incidence of adverse reactions to COVID-19 vaccines and worsening AD symptoms did not significantly differ between dupilumab-treated patients and the control group. The itch NRS score increased significantly after vaccination (*p* < 0.001). No serious adverse reactions were observed in patients with AD after COVID-19 vaccination. Exacerbation of pruritus and AD symptoms was observed but was mostly mild and transient [31,37].

### 4.3. Alopecia Areata

Alopecia areata (AA) is a common autoimmune disease characterized by non-scarring hair loss. There have been only a few reports of COVID-19 post-vaccination alopecia areata in recent years. The mechanism of this potential association is unclear but may involve the upregulation of proinflammatory cytokines such as interleukin (IL)-6, tumor necrosis factor (TNF)-α, and IFN-ɣ, which are also implicated in AA pathogenesis [38].

### 4.4. General

In a large prospective observational study by van Dam et al. including 2111 patients with IMIDs, 10.6% of the study population developed increased disease activity at 2–12 months following COVID-19 vaccination [39]. When categorized per IMID, increased disease activity within 60 days after the start of primary immunization was reported in 25/129 (19.4% [95% CI 13.2–27.5]) patients with myasthenia gravis and in 24/151 (15.8% [95% CI 10.6–22.8]) patients with inflammatory neuropathies or myopathies, which was more frequently, compared to 174/1830 (9.5% [95% CI 8.2–10.9]) in the other IMID categories combined, *p* < 0.001 and *p* = 0.01, respectively. In systemic lupus erythematosus, atopic dermatitis, and other dermatological diseases, self-reported exacerbations of disease activity following vaccination were less common [39].

## 5. Efficacy of COVID-19 Vaccines in Patients with IMIDs

Regarding the efficacy of COVID-19 vaccines in patients with IMIDs, Raptis et al. compared the humoral immunogenicity following mRNA vaccines in 565 patients with inflammatory rheumatic disease. The authors reported that monotherapy with a biological agent such as rituximab and TNF inhibitors was associated with reduced vaccine response [40]. In another study, response to the BioNTech/Pfizer vaccine was evaluated in 84 patients with IMIDs and 182 healthy controls [41]. The authors of this study reported that patients with IMID developed antibody response at a slower rate than controls, and a higher percentage of patients in the IMID group did not respond to the vaccine at all. This difference was observed even among patients with IMIDs who were not receiving any biologic or disease-modifying anti-rheumatic treatment, indicating that the underlying disease may be responsible for the differences noticed in response, rather than the type of treatment. Moreover, the immune dysregulation seen in HS, such as altered cytokine profiles and impaired immune cell function, raises concerns about the potential impact of HS on vaccine response. However, preliminary studies suggest that HS patients can mount an adequate immune response to COVID-19 vaccination, with comparable antibody levels to the general population [41].

Finally, Otten et al. evaluated 312 patients receiving biologic or non-biologic treatment for inflammatory bowel disease and found that seroconversion following COVID-19 vaccination was reduced in the patients who were on anti-TNF-a therapy. The reduced seroconversion in patients receiving biologics shown in some studies, may reflect the reduced immune response against COVID-19 vaccination among those patients. This may subsequently be the underlying reason why our patients receiving biologics experienced fewer HS flares, indicating a potential “protective” role of biologic therapy against HS flares [42].

The mRNA COVID-19 vaccines activate the innate immune system through recognition by pattern recognition receptors (PRRs), such as Toll-like receptor 7 (TLR-7) present on dendritic cells (DCs) and other innate immune cells. DCs play a critical role in initiating the immune response by recognizing mRNA and producing proinflammatory cytokines. These cytokines promote T helper 1 (Th1) cell activation, which is essential for a robust cellular immune response. However, in patients with IMIDs, this enhances activation may exacerbate underlying inflammatory pathways, potentially leading to increased disease activity. Additionally, other innate immune cells, such as macrophages, natural killer (NK) cells, and neutrophils, contribute to the inflammatory milieu by producing cytokines and chemokines, which can further influence autoimmune and inflammatory processes. Understanding the specific interactions between mRNA vaccines and the innate immune system is crucial for optimizing vaccination strategies and managing potential adverse effects in IMID patients [16,43,44].

The role of CD8+ T cells in immune-mediated inflammatory diseases (IMIDs) is critical, as these cells can exacerbate tissue damage and inflammation by targeting self-antigen-presenting cells. In diseases such as psoriasis, CD8+ T cells infiltrate affected tissues, contributing to disease pathogenesis and flares. The mRNA COVID-19 vaccines could elicit immune responses involving both CD4+ and CD8+ T cells. CD4+ T cells, particularly Th1 cells, produce proinflammatory cytokines (e.g., IFN-γ, TNF-α), which probably exacerbate inflammation in IMID patients. Meanwhile, CD8+ T cells contribute to disease flares by increasing cytotoxic activity and secreting cytokines like IFN-γ. Understanding the differential effects of vaccine-induced CD4+ and CD8+ T cell activation is essential for managing potential adverse effects in IMID patients [43,45].

One of the mechanisms by which COVID-19 vaccines could lead to the activation of autoreactive T cells that would then result in an immune-mediated inflammatory disease flare is molecular mimicry, whereby viral or microbial antigens resemble self-antigens. While still limited, with scarce direct evidence relating COVID-19 vaccines to molecular mimicry in the context of IMID flares, some studies have pointed out the sharing of homology between peptides in SARS-CoV-2 and human proteins. The molecular mimicry mechanism is plausible because of historical precedents from infections such as Epstein–Barr virus and coxsackievirus, which have been implicated in autoimmune conditions. This thereby gives the spike protein encoded by the COVID-19 mRNA vaccines, in which molecular mimicry with host proteins might take place in the induction of autoreactive T cells, leading to IMID flares in susceptible individuals [46,47,48,49].

Additionally, the lack of seroconversion or slow rate following COVID-19 vaccination may correlate with flares of IMIDs. This inadequate immune response could result in prolonged antigen presence, leading to sustained immune activation and inflammation, thereby triggering disease flares. Understanding the interplay between seroconversion rates and cytokine responses is crucial for managing potential adverse effects in IMID patients following vaccination [50,51,52].

Non-mRNA COVID-19 vaccines, including adenoviral vector and protein subunit vaccines, were generally well tolerated in patients with immune-mediated inflammatory diseases. Adenoviral vector vaccines, such as the AstraZeneca vaccine ChAdOx1-S and Johnson & Johnson’s have been linked to mild–moderate flares of similar magnitude to those reported with mRNA vaccines. Protein subunit vaccines like Novavax showed a good safety profile without any meaningful flare increment. It could be that different mechanisms of antigen presentation and innate immune activation are behind the higher flare rates observed with the mRNA vaccines compared to other vaccine types. mRNA vaccines undergo intracellular production, followed by antigen presentation through the MHC class I and II pathways, thus inducing robust activation of CD4+ and CD8+ T cells. Besides the mechanisms described above, mRNA vaccines are known to engage innate immunity via TLRs, which is associated with strong cytokine responses. These mechanisms might contribute to flare occurrence in susceptible individuals [2,53,54,55,56].

## 6. Concluding Remarks

The COVID-19 pandemic and the beginning of the vaccination campaign have raised challenges in the management of chronic inflammatory diseases. Physicians should be prepared to treat disease exacerbations following COVID-19 vaccines, since, as indicated from recent studies, about one out of five patients with HS may experience a flare following vaccination. Moreover, these flares should be anticipated more often following mRNA vaccination.

Understanding the relationship between COVID-19 vaccine administration and flares in individuals with IMIDs is crucial. This knowledge can greatly improve patient care and vaccination outcomes. By incorporating an evidence-based approach, healthcare professionals can adeptly manage the intricacies of vaccinating patients with chronic inflammatory diseases, thereby assuring the comprehensive advantages of vaccination while mitigating any possible adverse effects. This approach will result in better individual patient outcomes and broader public health objectives by increasing vaccination rates and reducing the overall COVID-19 impact.

Insights obtained from COVID-19 vaccination efforts will also be critical in preparing for upcoming public health threats, thus ensuring enhanced readiness for vulnerable population groups from new infectious diseases.

## Data Availability

No new data were created or analyzed in this study. Data sharing is not applicable to this article.
